# New and cryptic species of intertidal mites (Acari, Oribatida) from the Western Caribbean – an integrative approach

**DOI:** 10.1080/01647954.2018.1532458

**Published:** 2019-01-24

**Authors:** Tobias Pfingstl, Andrea Lienhard, Julia Baumann

**Affiliations:** a Institute of Biology, University of Graz, Graz, Austria

**Keywords:** Biogeography, Florida, morphometry, *Litoribates*, Panama, *Thalassozetes*

## Abstract

The present study highlights the distribution, systematics, morphology, genetics, and ecology of two newly discovered intertidal oribatid mites from the Western Caribbean. The fortuyniid *Litoribates floridae* sp. nov. represents a cryptic species as it looks nearly identical to *L. bonairensis*. The two species can be distinguished only by subtle morphological and morphometric characteristics, whereas cytochrome oxidase subunit I gene sequences clearly separate the two taxa. The absence of morphological divergence in these disjunct species may have resulted from stabilizing selection due to the extreme intertidal environment. *Litoribates floridae* sp. nov. is presently known from the Florida Keys, primarily in mangrove leaf litter. The selenoribatid *Thalassozetes balboa* sp. nov. can be distinguished from all known congeners by a unique cuticular notogastral pattern, the presence of only two pairs of adanal setae, and two ventral teeth on each leg claw. It is morphologically most similar to *T. barbara* from the Eastern Caribbean. *Thalassozetes balboa* sp. nov. was found in Panama and Florida. This species usually occurs on rocky substrate and feeds on the intertidal alga *Bostrychia*.

*Litoribates floridae*http://www.zoobank.org/urn:lsid:zoobank.org:act:A4B830FC-A03F-405D-9DE4-DE4C39DB6211

*Thalassozetes balboa*http://www.zoobank.org/urn:lsid:zoobank.org:act:EBF8C435-5C07-4B0E-8279-2101DC9E2CD4

## Graphical Abstract

**Figure:**
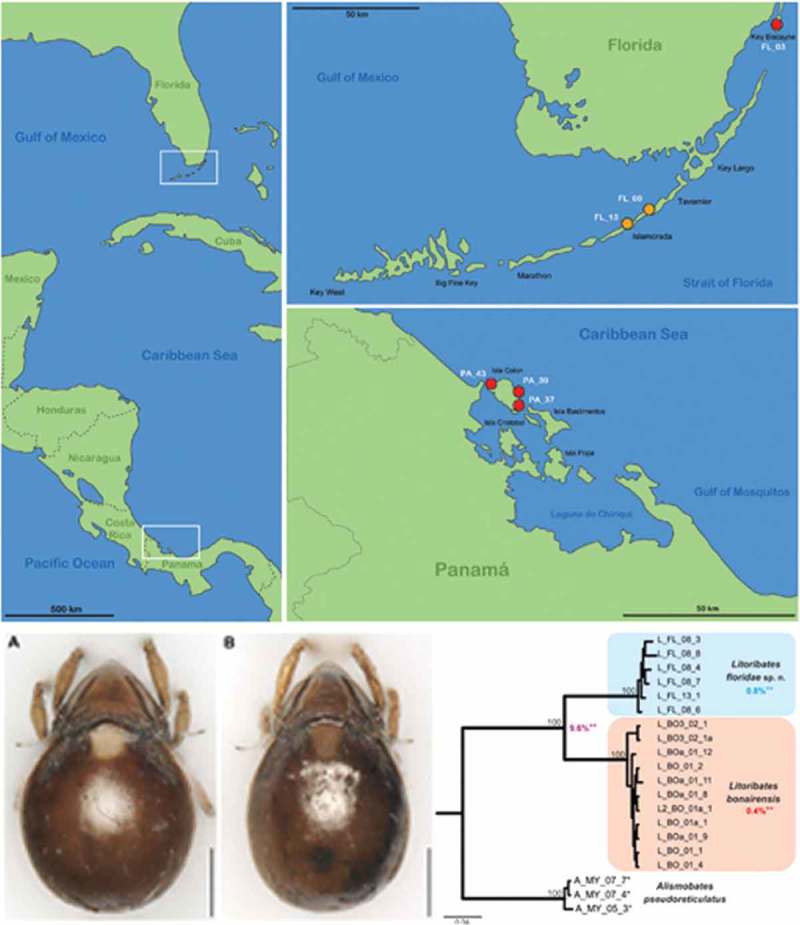

## Introduction

Fortuyniidae and Selenoribatidae are closely related oribatid mite families that are exclusively associated with intertidal habitats (Pfingstl 2017). They feed on littoral algae (Schuster 1966; Pfingstl 2013a) and use plastron respiration to breathe during daily tidal submergence (Pugh et al. 1990; Pfingstl and Krisper 2014). Despite their occurrence on the shores of every ocean, they are restricted to warmer climates and hence inhabit subtropical and tropical shores (Pfingstl and Schuster 2014). Presently, the family Fortuyniidae comprises four genera, *Alismobates* Luxton, 1992, *Circellobates* Luxton, 1992, *Fortuynia* Hammen, 1960, and *Litoribates* Pfingstl & Schatz, 2017, with 24 species in total. The family Selenoribatidae includes nine genera, *Arotrobates* Luxton, 1992, *Carinozetes* Pfingstl & Schuster, 2012, *Indopacifica* Pfingstl et al., 2018, *Psednobates* Luxton, 1992, *Rhizophobates* Karasawa & Aoki, 2005, *Schusteria* Grandjean, 1968, *Selenoribates* Strenzke, 1961, *Thalassozetes* Schuster, 1963, and *Thasecazetes* Pfingstl, Baumann, Lienhard & Schatz, 2017, with 28 species.

Most species in each family have an Indo-Pacific distribution (Pfingstl and Schuster 2014), and the Caribbean area remained unexamined for intertidal oribatid mites for a long time. Schuster (1977, 1989) was the first to record Selenoribatidae from the Lesser Antillean islands, Dominica, and St. Lucia, but specimens were identified only to family level. Over two decades later, Pfingstl (2013b) provided the first definitive Caribbean species record *Thalassozetes barbara* Pfingstl, 2013b from the island of Barbados. Soon after it became evident that intertidal mites may be a common part of the Caribbean fauna as occurrences of the fortuyniid genera *Alismobates* and *Fortuynia*, as well as the selenoribatid *Carinozetes* from Barbados, Jamaica, and the Dominican Republic were reported (Pfingstl and Schuster 2014). Pfingstl et al. (2016) published further reports of *T. barbara* and new records of *Carinozetes mangrovi* Pfingstl, Lienhard & Jagersbacher-Baumann, 2014 and *Alismobates inexpectatus* Pfingstl & Schuster, 2012 from the Greater Antillean landmass of Hispaniola. Newly discovered Caribbean species have included *Schusteria marina* Pfingstl & Lienhard, 2017 from the coasts of Grenada and Martinique (Pfingstl and Lienhard 2017), *Litoribates bonairensis* Pfingstl, Baumann, Lienhard & Schatz, 2017, and *Thasecazetes falcidactylus* Pfingstl, Baumann, Lienhard & Schatz, 2017, the latter representing a newly erected genus, from the island of Bonaire (Pfingstl et al. 2017).

These recent records demonstrate that the Caribbean intertidal oribatid mite fauna is probably highly diverse and still poorly known. In the course of ongoing studies in this region, further *Thalassozetes* and *Litoribates* specimens were found on the coasts of Panama and Florida, respectively. The *Thalassozetes* individuals showed obvious morphological deviations from *T. barbara*, which occurs in the Eastern Caribbean (Pfingstl et al. 2016), whereas the *Litoribates* specimens from Florida exhibited a remarkable similarity to *L. bonairensis*, known from far distant Bonaire. Therefore, the aims of the present paper are (1) to assess the taxonomic identity of these specimens with morphological and molecular genetic means, (2) to describe new taxa in detail and discuss their morphological peculiarities, and (3) to provide their distribution patterns.

## Materials and methods

Samples of intertidal algae were scraped from rocks with a knife and decaying mangrove litter was collected during low tide. Algae and litter were put in Berlese-Tullgren funnels for about 24 h to extract mites, which were then stored in ethanol (100%) for morphological and molecular genetic investigation.

### Sample locations (Figure 1)

Coordinates for locations of individuals used only for morphological studies are provided in this section; coordinates for locations of individuals used for both morphological and molecular genetic studies are given in Table 1.

**Table 1.** GenBank accession numbers for COI and 18S rRNA gene sequences comprising all specimens included in genetic investigations. Sequences generated in this study appear in bold.

			GenBank Accession Nr.	
Species	Sample ID	Coordinates	COI	18S
*Thalassozetes balboa* sp. nov.	T_PA_37_3	9.415057°N,82.330787°W		MK035018
	T_PA_43_3	9.370821°N,82.239908°W		MK035019
*Thalassozetes barbara*	T_BA_30_1	13.212719°N,59.517116°W		MK035020
*Litoribates floridae* sp. nov.	L_FL_08_3	24.914264°N, 80.64089°W	MK035001	MK035021
	L_FL_08_4		MK035002	
	L_FL_08_6		MK035003	
	L_FL_08_7		MK035004	
	L_FL_08_8		MK035005	
	L_FL_13_1	24.890059°N,80.674575°W	MK035006	MK035022
*Litoribates bonairensis*	L_BO_01_1	12.103654°N,68.221720°W	MK035007	MF997503^d^
	L_BO_01_2		MK035008	MF997502^d^
	L_BO_01_4		MK035009	
	L_BOa_01_1		MK035010	
	L_BO3_02_1		MK035011	
	L_BO3_02_1^a^		MK035012	
	L_BOa_01_11		MK035013	
	L_BOa_01_12		MK035014	
	L_BOa_01_8		MK035015	
	L_BOa_01_9		MK035016	
	L2_BO_01a_1		MK035017	
*Alismobates pseudoreticulatus*	A_MY_07_7		MH285613^a^	MH285696^a^
	A_MY_05_3		MH285597^a^	
	A_MY_07_4		MH285610 ^a^	
*Alismobates reticulatus*				AB818526.1^b^
*Aquanothrus sp.*				KX397627.1^c^
*Fortuynia longiseta*				MH285693^a^
*Fortuynia rotunda*				AB818525.1^b^
*Fortuynia* sp.				MH285695^a^
*Fortuynia smiti*				MH285694^a^
*Hydrozetes confervae*				AB818523.1^b^
*Hydrozetes lemnae*				KX397632.1^c^
*Indopacifica pantai* (1)				MH285692^a^
*Indopacifica pantai* (2)				MH285691^a^
*Indopacifica parva*				MH285690^a^
*Schusteria littorea*				HM070345.1^e^
*Thasecazetes falcidactylus*				MF997501^d^
*Thalassozetes shimojanai*				AB818524.1^b^

^a^Pfingstl et al. (2018); ^b^Iseki & Karasawa (2014); ^c^Krause et al. (2016); ^d^Pfingstl et al. (2017); ^e^Pepato et al. (2010).

**Figure 1.**
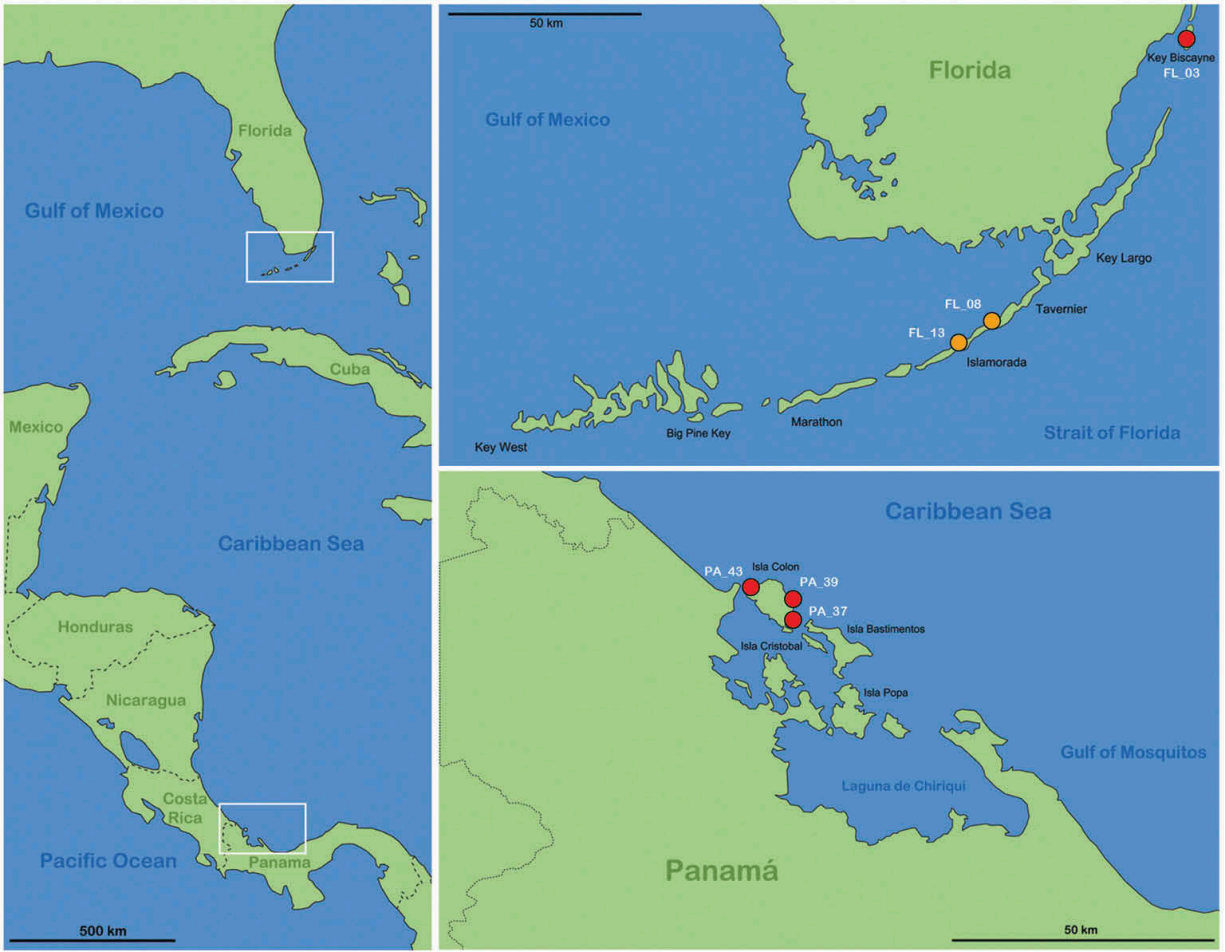
Maps illustrating the records of new species in the western Caribbean. Left vertical map provides an overview of the area with white frames indicating the position of the regions given in the two horizontal maps. Red circles represent records of *Thalassozetes balboa* sp. nov. and orange circles identify occurrence of *Litoribates floridae* sp. nov.; codes in white refer to the sampling location given in the Materials and Method section.

*Thalassozetes balboa* sp. nov.: Panama, Bocas del Toro, Isla Colon (1) Paunch (PA_37), intertidal algae (*Bostrychia*) from rock, 7 February 2017; (2) Bluff Beach (PA_39), coordinates 9.385454°N 82.23524°W, various intertidal algae from rock; 7 February 2017 (3) Boca del Drago (PA_43); intertidal algae (*Bostrychia*) from rock; 8 February 2017, all Panamanian samples collected by T. Pfingstl and A. Lienhard. Florida, U.S.A. (4) Key Biscayne (FL_03), coordinates 25.677296°N 80.164818°W, *Bostrychia* from mangrove roots (*Rhizophora mangle*); 12 February 2017, coll. T. Pfingstl and A. Lienhard.

*Litoribates floridae* sp. nov.: (1) Florida U.S.A, Florida Keys, Islamorada (FL_08); leaf litter under red mangrove (*Rhizophora mangle*); 13 February 2017, coll. T. Pfingstl and A. Lienhard. (2) Florida Keys, Indian Key Fill (FL_13); algae on rocks under red mangrove (*Rhizophora mangle*); 13 February 2017, coll. T. Pfingstl and A. Lienhard.

### Morphometric analyses

For morphometric investigations, specimens were placed in lactic acid (temporary slides) and measured using a compound light microscope (Olympus BH-2) and ocular micrometer.

**Figure 2.**
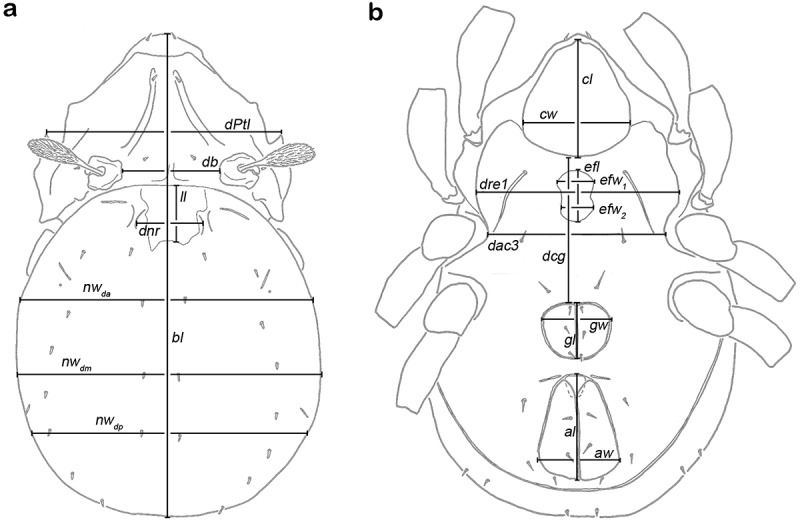
Graphic illustration of measured continuous variables shown on simplified drawings of *Thalassozetes barbara*. (a) Dorsal aspect: *bl –* body length, *dPtI –* distance between pedotecta I, *db –* distance between bothridia, *ll –* lenticulus length, *dnr –* distance notogastral ridges, *nw_da_* – notogastral width on level of seta *da, nw_dm_* – notogastral width on level of seta *dm, nw_dp_* – notogastral width on level of seta *dp*. (b) Ventral aspect: *cl –* camerostome length, *cw –* camerostome width, *dre1 –* distance between ridges on epimeron 1, *efl –* epimeral fovea length, *efw_1_* – epimeral fovea width anterior part, *efw_2_* – epimeral fovea width posterior part, *dcg –* distance between camerostome and genital orifice, *dac3 –* distance between acetabula 3, *gl –* genital orifice length, *gw –* genital orifice width, *al –* anal opening length, *aw –* anal opening width.

For the study of intraspecific variation, 20 continuous variables (Figure 2) were measured in 90 *T*. *balboa* sp. nov. individuals from three different populations in Panama (PA_37, PA_39, and PA_43). For species discrimination, 15 continuous variables (Pfingstl and Baumann 2017) were measured in 16 *L*. *floridae* sp. nov. specimens and compared to those of 19 *L. bonairensis* from Bonaire (data for the latter from Pfingstl et al. 2017). The *T. balboa* specimens from Panama were compared with 19 *T. barbara* individuals from Barbados.

Univariate statistics were performed for the species of each genus: Mean, minimum, maximum, standard deviation, and coefficient of variation (cv) were calculated to assess variation within and between species. The Mann–Whitney *U* test was used for comparing the means of variables between the species.

In the subsequent multivariate analyses, males and females were coded with different symbols in order to detect possible sexual dimorphism in the studied populations. To reveal patterns of morphometric variation, we conducted Principal Component Analyses (PCAs) on ln(x+1) transformed raw and size-corrected data of *Thalassozetes* and *Litoribates*. Size correction was performed by dividing each variable through the geometric mean of the respective specimen, as described in Pfingstl et al. (2017).

A linear discriminant analysis (LDA) was performed on raw and size-corrected data to reveal possible differences and to determine the most important variables differentiating *Thalassozetes* from Panama and Barbados and *Litoribates* from Bonaire and Florida. The performance of the classification by LDA was tested by calculating the percentage of specimens correctly classified by all-samples LDA and by leave-one-out cross-validation LDA. For testing the equality of means of populations, Bonferroni-corrected Hotelling’s T^2^ tests were used.

All analyses were performed with PAST 3.11 (Hammer et al. 2001).

### Molecular genetic analyses

In total, 20 specimens of *Litoribates* spp. and *Thalassozetes* spp. collected in Barbados, Bonaire, Florida, and Panama were analysed (see Table 1). Total genomic DNA was extracted from single individuals preserved in absolute ethanol. Extraction was carried out using the Chelex method (Casquet et al. 2012) with the following adjustments: whole specimens were crushed against the tube wall in microcentrifuge tubes containing 55 µl of a 10% Chelex solution and were extracted for 20 min at 95°C. Two gene fragments were sequenced for this study: the mitochondrial cytochrome oxidase subunit I (*COI*) and the nuclear *18S* rRNA (*18S*). A 567 bp fragment of the *COI* gene was amplified using the primer pairs Mite COI-2F and Mite COI-2R (Otto and Wilson 2001). The complete *18S* rRNA (~1.8 kb) was amplified according to the PCR protocol of Dabert et al. (2010) using the recommended primers (Skoracka and Dabert 2010). PCR conditions for the *COI* gene fragment are given in Pfingstl et al. (2014). DNA purification (with the enzyme cleaner ExoSAP-IT, Affymetrix; and the Sephadex G-50 resin, GE Healthcare) and sequencing steps (using the BigDye Sequence Terminator v3.1 Cylce Sequencing Kit, Applied Biosystems) were conducted according to the methods published by Schäffer et al. (2008). Sequencing was performed in both directions on an automated capillary sequencer (ABI PRISM 3130xl, Applied Biosystems).

Alignments were generated by means of the program MEGA6 (Tamura et al. 2013). For both gene fragments, Bayesian 50% majority rule consensus trees were generated with MrBAYES 3.1.2 (Ronquist and Huelsenbeck 2003) applying a MC^3^ simulation with 20 million generations (5–7 chains, 2 independent runs, 10% burn-in, GTR+I+G model). Results were analysed in TRACER v.1.6 (Rambaut and Drummond 2007) to check for convergence and to ensure the stationarity of all parameters. Uncorrected *p*-distances were calculated in MEGA6. All sequences obtained from this study were deposited in GenBank (www.ncbi.nlm.nih.gov/genbank; accession numbers for *COI*: MK035001–MK035017, *18S*: MK035018–MK035022; Table 1). For the *18S* data set, all already-published fortuyniid and selenoribatid sequences were integrated in the alignment, and as there is no 18S sequence data from closely related families as for example Ameronothridae or Podacaridae, *Hydrozetes* served as closest related outgroup. For the COI, data set sequences of *Alismobates pseudoreticulatus* served as outgroup.

### Drawings and photographs

Preserved animals were embedded in Berlese mountant for microscopic investigation in transmitted light. Drawings were made with an Olympus BH-2 Microscope equipped with a drawing attachment. These drawings were first scanned, then processed, and digitized with the free and open-source vector graphics editor Inkscape (https://inkscape.org).

For photographic documentation, specimens were air-dried and photographed in reflected light with a Keyence VHX-5000 digital microscope.

Morphological terminology used in this paper follows that of Grandjean (1953) and Norton and Behan-Pelletier (2009).

## Results

### Descriptions of new taxa

**Family Fortuyniidae** Hammen, 1963

**Genus *Litoribates*** Pfingstl & Schatz, 2017

Type species – *Litoribates caelestis* Pfingstl & Schatz, 2017

***Litoribates floridae*** sp. nov.

#### Type material/locality

Holotype: adult female, Florida U.S.A, Florida Keys, Islamorada (FL_08), leaf litter under red mangrove (*Rhizophora mangle*); 13 February 2017, coll. T. Pfingstl and A. Lienhard, preserved in ethanol, deposited at the Naturhistorisches Museum Wien, Vienna. Four paratypes from the same sample, deposited in the collections of the US National Museum and the Senckenberg Museum für Naturkunde Görlitz, respectively. Additional non-type specimens are stored in the collections of the Institute of Biology, University of Graz.

#### Etymology

The specific epithet is a noun in the genitive case, referring to Florida, the type locality of this species.

#### Diagnosis

Adult instar brown sclerotized. Length 336–375 µm, width 231–259 µm. Notogaster rounded, almost circular in dorsal view. Slender lamellar ridges anteriorly converging. Sensillus slightly clavate and spinose at tip. Cerotegument finely granular, obviously larger granules in humeral areas. Irregular longitudinal cuticular ridges flanking lenticulus, conspicuous cuticular pattern absent in other notogastral areas. Fourteen pairs of setiform, notogastral setae. Epimeral setation 3-1-2-2, setae *1b* and *3b* the longest.

#### Remarks

Presently, there are only three species of this genus known. *Litoribates floridae* can be easily distinguished from the Pacific *L. caelestis* by the absence of the obvious reticulate cuticular surface pattern present in the latter and its larger size (mean length 360 µm vs. 322 µm). It is morphologically very similar with *L. bonairensis* but has several irregular longitudinal cuticular ridges in the humeral region that are either absent or only faintly developed in the latter species.

#### Description

##### Measurements

Females (N = 9), length: 336–375 μm (mean 368 μm), width: 240–259 μm (mean 249 μm); males (N = 7), length: 350–363 μm (mean 357 μm), width: 231–246 μm (mean 238 μm).

##### Integument

Colour dark brown.

##### Prodorsum

**Figure 3.**
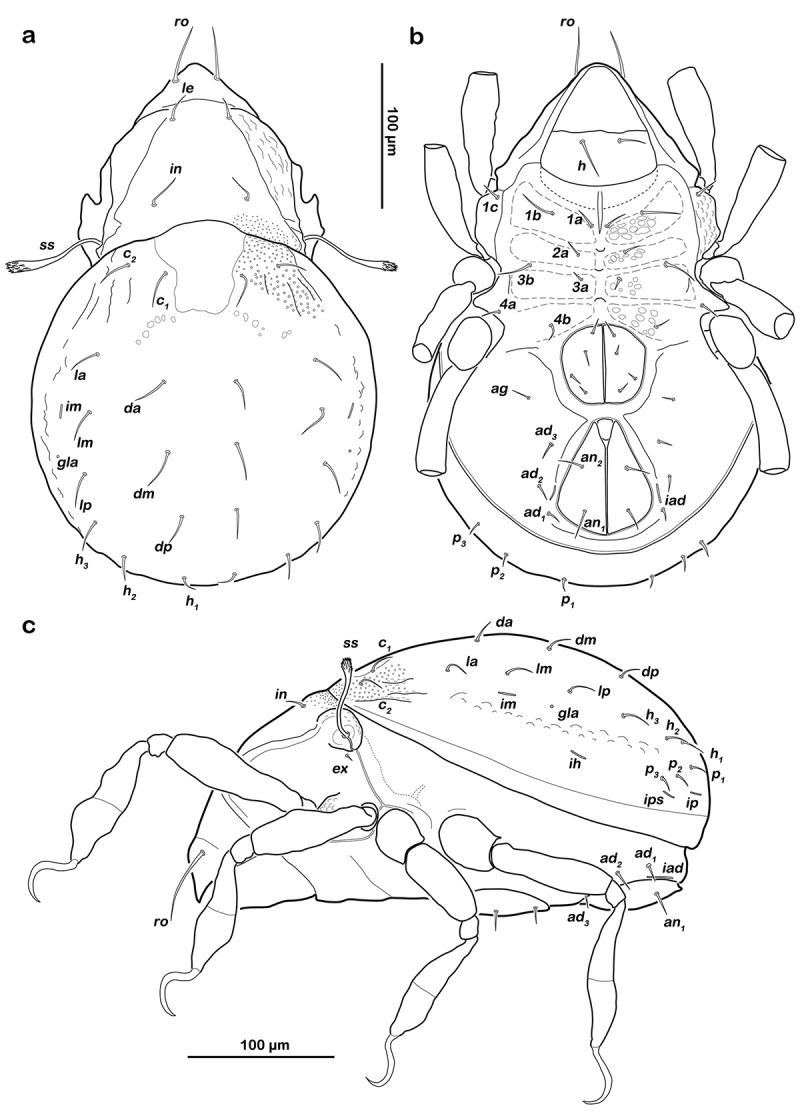
*Litoribates floridae* sp. nov. adult (a) dorsal view; (b) ventral view, distal leg segments omitted; (c) lateral view.

**Figure 4.**
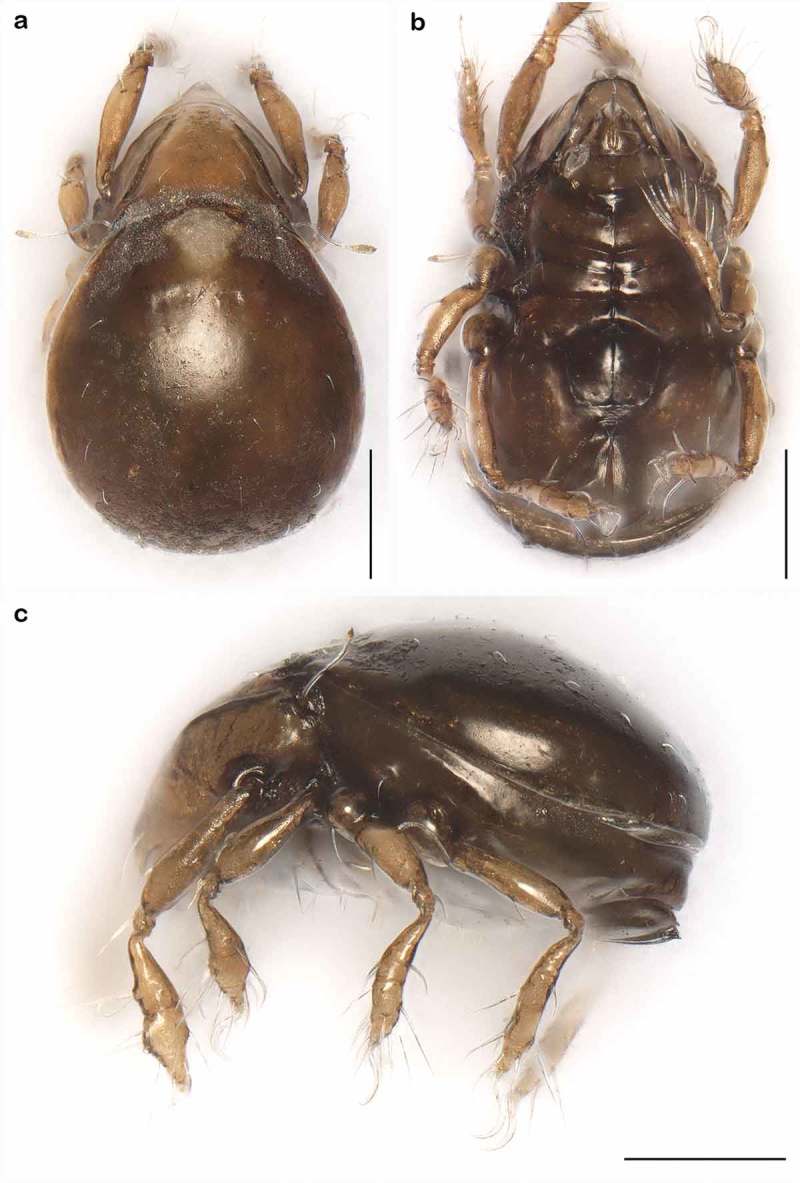
Photographs (stacked stereomicroscopic images) of *Litoribates floridae* sp. nov. adult (a) dorsal view; (b) ventral view; (c) lateral view. Scale bars 100 µm.

(Figures 3(a) and 4(a)) Cerotegument finely granular, larger granules next to anterior notogastral border and bothridia. Rostrum nearly triangular in dorsal view, projecting anteroventrally in lateral view; demarcated from remainder of prodorsum by faint transverse ridge. Pair of converging, slender lamellar ridges in slightly lateral position, reaching from bothridium to lamellar seta. Rostral seta (*ro*) robust, setiform, smooth (approx. 30 µm). Lamellar seta (*le*) setiform, short (approx. 15 µm), and smooth. Interlamellar seta (*in*) setiform (approx. 20 µm), exobothridial seta (*ex*) minute. Bothridium hardly protruding, borders not clearly defined; orifice narrow and circular. Sensillus (*ss*) long (approx. 75 µm), slightly bent caudally, slightly clavate, and spinose at the tip.

##### Gnathosoma

**Figure 5.**
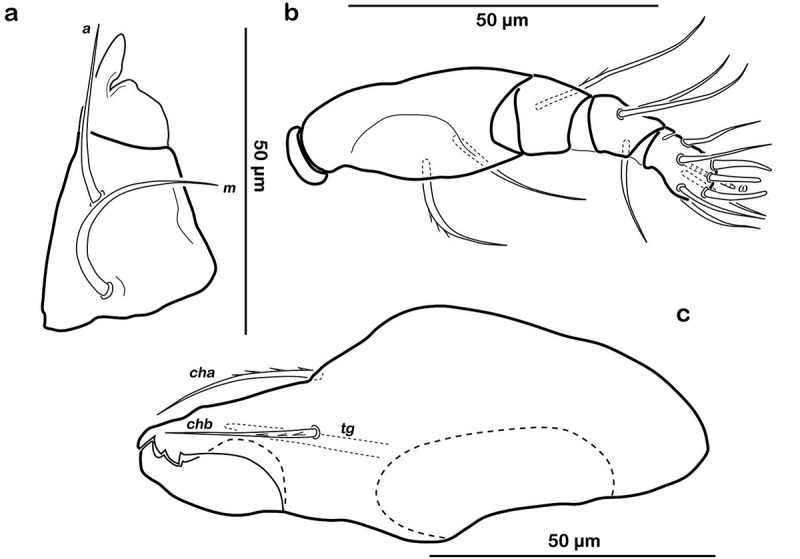
*Litoribates floridae* sp. nov. adult mouthparts (a) right rutellum, ventral view; (b) left pedipalp, paraxial view; (c) left chelicera, antiaxial view.

Typical for the genus. Distal part of rutellum developed as thin triangular membrane, slightly curved inward with longitudinal incision (Figure 5(a)). Setae *a* and *m* long (approx. 30 µm), robust, and smooth. Mentum regular, seta *h* setiform, thin (approx. 20 µm). Palp setation 0-2-1-3-8 (solenidion not included), trochanter very short, femur by far longest segment, genu, tibia, and tarsus of almost equal length (Figure 5(b)). Chelicera with mobile digit darker sclerotized; distinct teeth interlocking. Träghård’s organ (*tg*) slender blunt finger-like oncophysis. Seta *cha* and *chb* dorsally slightly pectinate both of the same length (approx. 25 µm) (Figure 5(c)).

##### Gastronotic region

(Figures 3(a) and 4(a)) Conspicuously rounded in dorsal view, convex in lateral view; anterior notogastral margin distinct, forming a slightly overhanging bulge in area of lenticulus. Lenticulus slightly trapezoid with slightly irregular borders. Cerotegument finely granular, obviously larger granules in humeral areas next to lenticulus. Irregular short longitudinal cuticular ridges flanking lenticulus. Several small circular light spots posterior to seta *c_1_*, representing sigilla of dorsoventral muscles. Fourteen pairs of setiform notogastral setae (8–16 µm), *c_1_-_2_, da, dm, dp, la, lm, lp, h_1_-_3_, p_1-3_*; seta *c_3_* absent. Five pairs of notogastral lyrifissures present; *ia* laterad of seta *c_2_*, hardly discernible due to strong cerotegumental granulation and cuticular ornamentation; *im* laterad of seta *lm; ih* laterad and anterior to *h_3_*; lyrifissures *ip* and *ips* laterally of seta *p_3_* and *p_2_*, respectively. Orifice of opisthonotal gland (*gla*) laterad, between seta *lm* and *lp*.

##### Lateral aspect

(Figures 3(c) and 4(c)) Cerotegument mostly finely granular, larger granules on pedotectum I and in areas surrounding acetabula. Pedotectum I small rounded, slightly projecting. Pedotectum II absent. Discidium present, not conspicuously expressed. Van der Hammen’s organ present; typical for the genus (for details, see Pfingstl and Schatz 2017).

##### Podosoma and venter

(Figures 3(b) and 4(b)) Cerotegument finely granular. Epimeral setation 3-1-2-2, all setae setiform and smooth. Setae *1b* and *3b* longest (approx. 18 µm), others shorter (10 µm). Internal borders of all epimera well visible. Median longitudinal small ridge on epimeron I, other sternal surface lines slightly protruding with small indentations at each transition from one epimeral segment to the other. Genital and anal opening closely adjacent, both surrounded by slightly darker cuticle. Rounded genital plates with four pairs of fine setae (approx. 10 µm). One pair of setiform aggenital setae *ag*. Anal valves strongly triangular. Outer part of preanal organ rectangular with rounded edges, inner part shaped like transverse bar. Two pairs of short anal setae, *an_1-2_* (approx. 10 µm). Three pairs of short adanal setae, *ad_1-3_* (approx. 8 µm). Lyrifissure *iad* flanking posterior third of anal plates.

##### Legs

**Figure 6.**
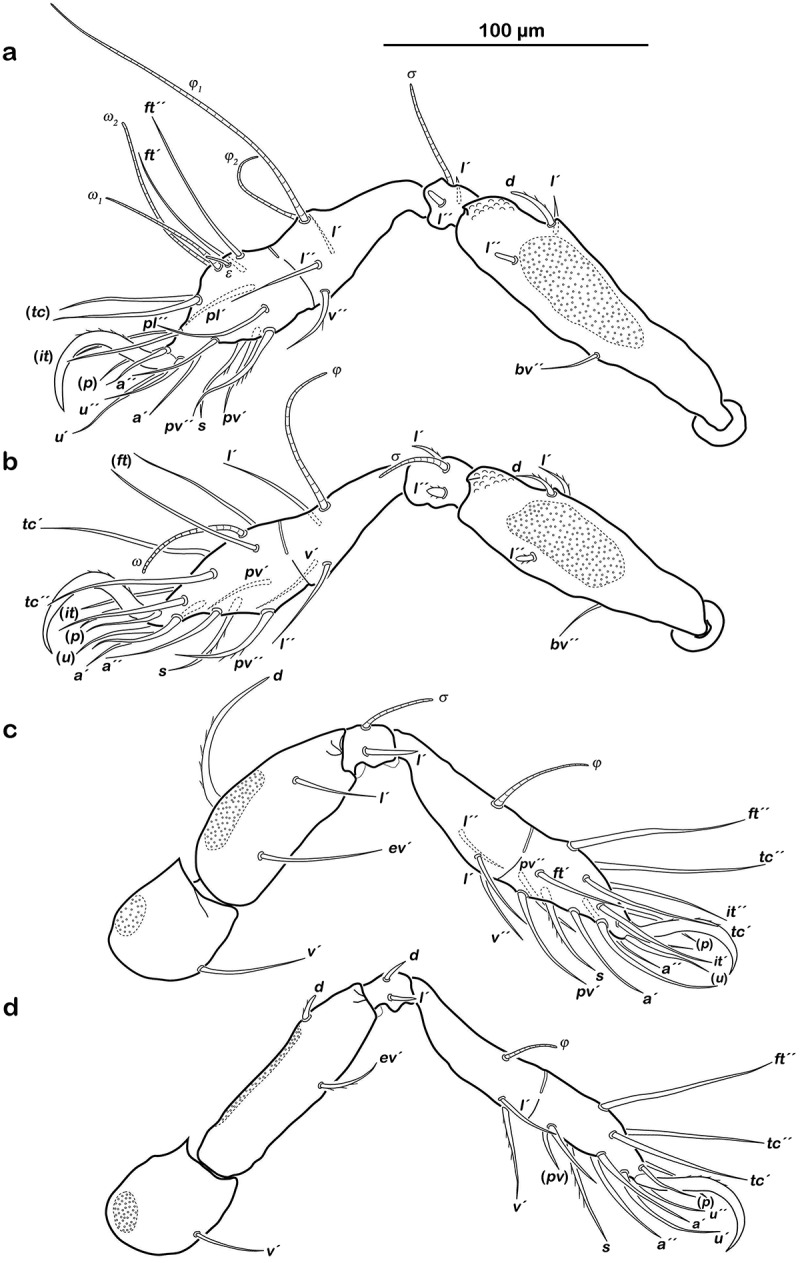
*Litoribates floridae* sp. nov. adult left legs, antiaxial view (a) leg I; (b) leg II; (c) leg III; (d) leg IV.

(Figure 6) Monodactylous and slender. Long, strong hook-like claws, with inconspicuous dorsal dentition. Cerotegument generally finely granular, larger granules only on distal third of all femora. Trochanter III and IV with obvious dorsal spur. All genua with ventral transversal ridge. Large elongate porose areas on ventral paraxial side of femora I and II and on paraxial dorsal aspect of femora III and IV. Kidney-shaped porose areas on paraxial dorsal aspect of trochanters III and IV. Dorsal seta *d* on all femora slightly thickened and barbed on outer curvature. Lateral setae of femora and genua I and II blunt, short, broadened, and slightly serrate. Ventral setae of all tibiae and tarsi long and slightly serrate ventrally. Tibia IV with two setae. Solenidia *φ_1-2_* on tibia I borne on vaguely delimited mound. Chaetome and solenidia given in Table 2.

**Table 2.** Leg chaetome and solenidia for the new species.

	Trochanter	Femur	Genu	Tibia	Tarsus	Chaetome	Solenidia
*Litoribates floridae* sp. nov.							
Leg I	–	*d, bv´´*, (*l*)	(*l), σ*	(*l), v´´, φ_1_, φ_2_*	(*pl*), (*pv), s*, (*a*), (*u*), (*p*), (*tc*), (*ft*), (*it), ε, ω_1_, ω_2_*	0-4-2-3-18	1-2-2
Leg II	–	*d, bv´´*, (*l*)	(*l*), *σ*	(*l*), *v´, φ*	(*pv*), *s*, (*a*), (*u*), (*p*), (*tc*), (*ft*), (*it), ω*	0-4-2-3-15	1-1-1
Leg III	*v´*	*d, l´, ev´*	*l´, σ*	(*l), v*´´, *φ*	(*pv), s*, (*a*), (*u*), (*p*), (*tc*), (*ft*), (*it*)	1-3-1-3-15	1-1-0
Leg IV	*v´*	*d, ev´*	*d, l´*	*l´, v*´, *φ*	(*pv), s*, (*a*), (*u*), (*p*), (*tc), ft*´´	1-2-2-2-12	0-1-0
*Thalassozetes balboa* sp. nov.							
Leg I	–	*d*, (*l*)	(*l), σ*	(*l), v´, φ_1_, φ_2_*	(*pl*), (*pv), s*, (*a*), (*u*), (*p*), (*tc*), (*ft*), (*it), ε, ω_1_, ω_2_*	0-3-2-3-18	1-2-2
Leg II	–	*d*, (*l*)	(*l*), *σ*	(*l*), *v´, φ*	(*pv), s*, (*a*), (*u*), (*p*), (*tc*), (*ft*), (*it), ω*	0-3-2-3-15	1-1-1
Leg III	*v´*	*d, ev´*	*l´, σ*	*l´, v*´´, *φ*	(*pv), s*, (*a*), (*u*), (*p*), (*tc*), (*ft*)	1-2-1-2-13	1-1-0
Leg IV	*v´*	*d, ev´*	*l´*	*l´*, (*v), φ*	(*pv), s*, (*a*), (*u*), (*p*), (*tc), ft*´´	1-2-1-3-12	0-1-0

() = pairs of setae.

**Family Selenoribatidae** Schuster 1963

**Genus *Thalassozetes*** Schuster, 1963

Type species – *Thalassozetes riparius* Schuster, 1963

***Thalassozetes balboa*** sp. nov.

#### Type material/locality

Holotype: adult female preserved in ethanol, Panama, Bocas del Toro, Isla Colon, Paunch (PA_37), intertidal algae (*Bostrychia*) from rock, 7 February 2017, deposited at the Naturhistorisches Museum Wien. Four paratypes preserved in ethanol: adult females; Panama, Bocas del Toro, Isla Colon, Boca del Drago (PA_43), intertidal algae (*Bostrychia*) from rock, deposited at Museo de Invertebrados Fairchild, Universidad de Panamá. Additional non-type specimens from all locations are stored in the collections of the Institute of Biology, University of Graz.

#### Etymology

As this species was found in Panama and Florida, the name was chosen to somehow relate to both locations. The specific epithet “*balboa*” (given as noun in apposition) refers to the Spanish explorer Vasco Núñez de Balboa; he led the first European expedition that crossed the Isthmus of Panama in 1513 and the Panamanian currency is named after him. It also refers to Rocky Balboa, a well-known movie character, a descendant of immigrants who made his fortune in the USA. The new species similarly may be an immigrant that has now successfully settled in North America. Moreover, *T. balboa* sp. nov. was predominantly found on “rocky” substrate.

#### Diagnosis

Dark brown sclerotized mites. Length 277–312 µm, width 154–194 µm. Notogaster oval in dorsal view with characteristic reticulate-foveate pattern. Lamellar ridges present. Sensillus clavate, distal half spinose. Pair of weak anterior notogastral ridges present, mediad of elliptic depressions. Fourteen pairs of setiform, notogastral setae. Median sternal depression on epimeron I, shaped like inverted pear. Three pairs of genital setae, two pairs of adanal and anal setae. Lyrifissure *iad* oblique, next to anterior corner of anal orifice. Legs monodactylous, claws with two ventral teeth.

#### Remarks

*T. balboa* sp. nov. can be distinguished from the other three *Thalassozetes* species by its specific reticulate-foveate cuticular pattern, with the centre of notogaster covered with regularly distributed conglomerates of irregular circular or elliptic depressions, fading laterally and caudally in smaller foveate pattern; by two pairs of adanal setae instead of three; and by having two ventral teeth on each claw instead of one.

#### Description

##### Measurements

Females (N = 26), length: 280–312 μm (mean 303 μm), width: 172–194 μm (mean 182 μm); males (N = 64), length: 277–305 μm (mean 289 μm), width: 154–182 μm (mean 170 μm).

##### Integument

Colour dark brown.

##### Prodorsum

**Figure 7.**
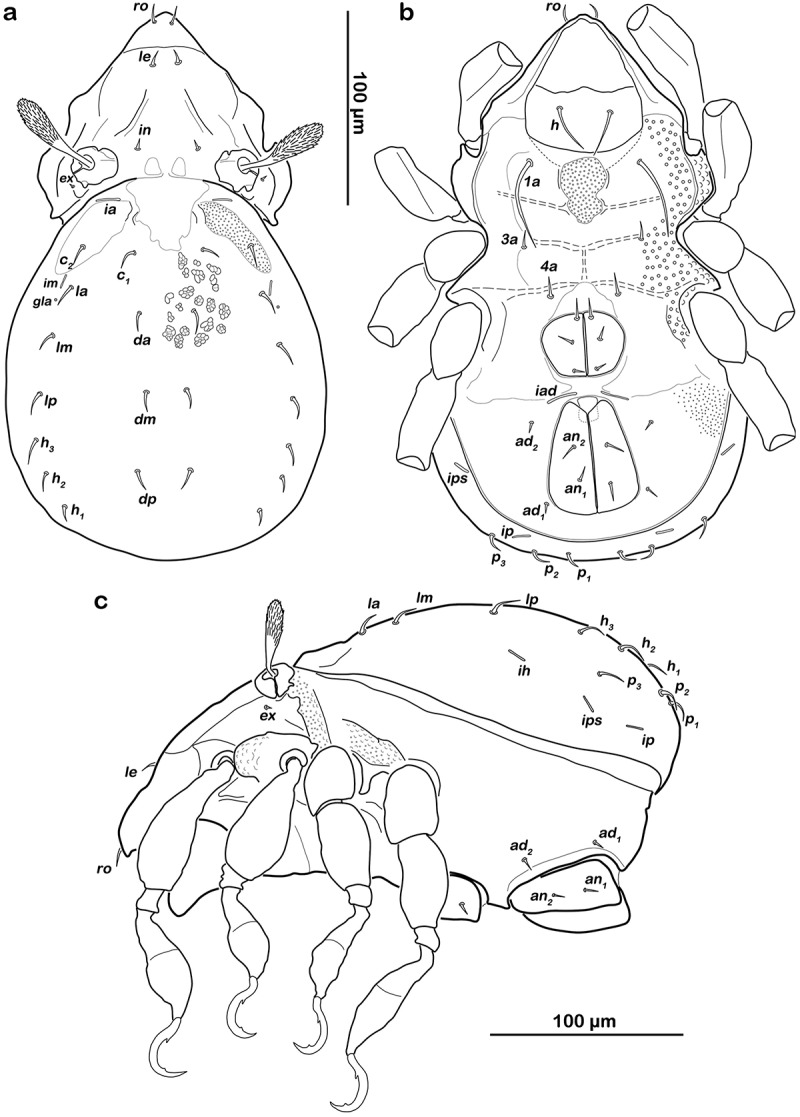
*Thalassozetes balboa* sp. nov. adult (a) dorsal view; (b) ventral view, distal leg segments omitted; (c) lateral view.

**Figure 8.**
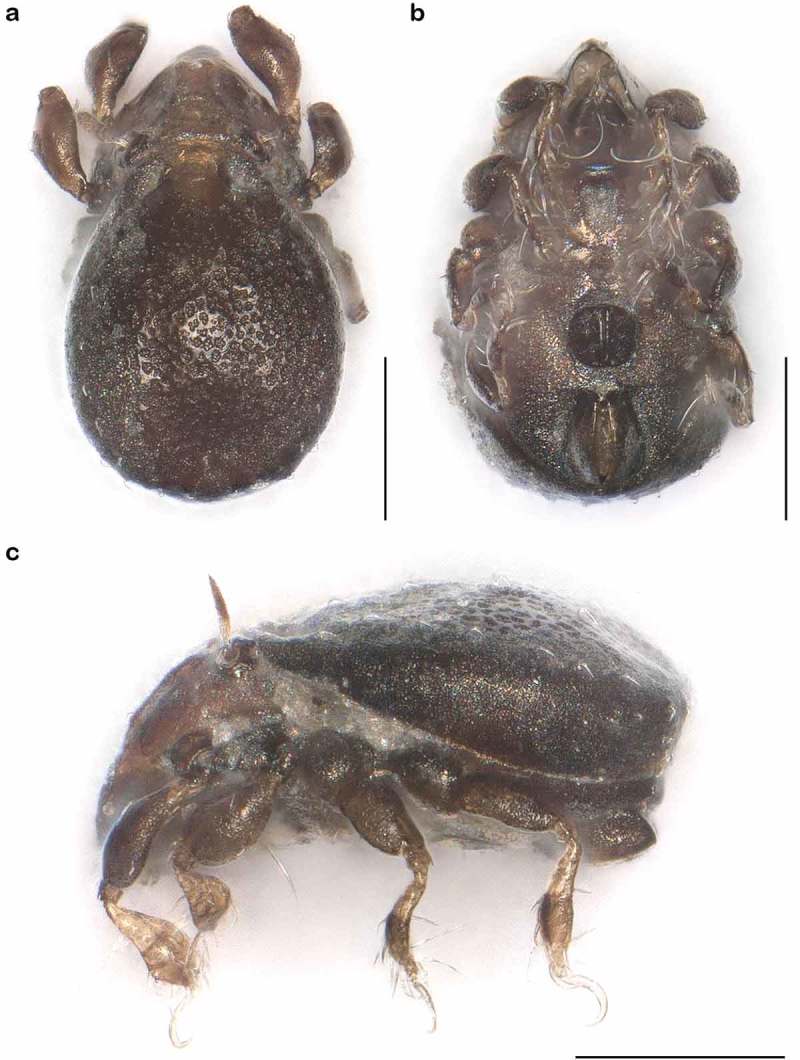
Photographs (stacked stereomicroscopic images) of *Thalassozetes balboa* sp. nov. adult (a) dorsal view; (b) ventral view; (c) lateral view. Scale bars 100 µm.

(Figures 7(a) and 8(a)) Cerotegument finely granular. Rostrum nearly triangular in dorsal view, projecting anteroventrally in lateral view. Rostrum demarcated from remainder of prodorsum by faint transverse ridge. Pair of inward-curving, slender lamellar ridges, reaching from bothridium to lamellar seta. Rostral seta (*ro*) and lamellar seta (*le*) short, setiform, smooth (approx. 5–6 µm). Interlamellar seta (*in*) short and setiform (approx. 5 µm), exobothridial seta (*ex*) minute. Bothridium large and strongly protruding, orifice circular. Sensillus of normal length (approx. 55 µm), clavate, distal half spinose.

##### Gnathosoma

**Figure 9.**
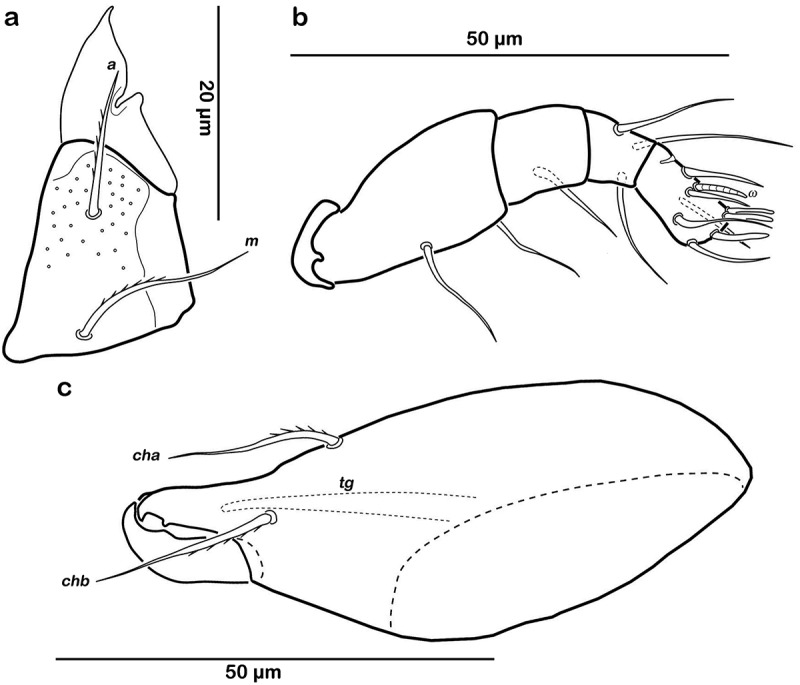
*Thalassozetes balboa* sp. nov. adult mouthparts (a) right rutellum, ventral view; (b) left pedipalp, paraxial view; (c) left chelicera, antiaxial view.

Typical for genus. Distal part of rutellum developed as thin triangular membrane, slightly curved inward with obvious longitudinal incision (Figure 9(a)). Setae *a* and *m* robust and slightly barbed unilaterally (approx. 15 µm). Mentum regular, seta *h* setiform, robust (approx. 20 µm). Palp setation 0-2-1-3-8 (solenidion not included), trochanter very short, femur longest segment, genu, tibia and tarsus of almost equal length (Figure 9(b)). Cheliceral digits with two teeth. Seta *cha* dorsally and *chb* laterally slightly pectinate, both setae of the same length (approx. 25 µm) (Figure 9(c)). Träghård’s organ (*tg*) slender blunt finger-like oncophysis.

##### Gastronotic region

(Figures 7(a) and 8(a)) Oval in dorsal view, convex in lateral view; dorsosejugal suture incomplete. Nearly triangular clear spot (vestigial lenticulus) with irregular borders on anterior notogastral median area. Cuticle with specific reticulate-foveate pattern, centre of notogaster covered with regularly distributed conglomerates of irregular circular or elliptic depressions, fading laterally and caudally in smaller foveate pattern. Pair of weak and highly variable (in shape) concave notogastral ridges framing light spot. Laterad of ridges, elliptic depressions lined with fine and densely packed granules. Fourteen pairs of setiform notogastral setae (approx. 10 µm), *c_1-2_, da, dm, dp, la, lm, lp, h_1-3_, p_1-3_*; seta *c_3_* absent. Five pairs of notogastral lyrifissures present; *ia* in humeral area adjacent to anterior border of elliptic cavity; *im* laterad of seta *la; ih* laterad and anterior to *h_3_*; lyrifissures *ip* and *ips* laterally of seta *p_3_* and *p_2_* respectively. Orifice of opisthonotal gland (*gla*) laterad between seta *la* and *lm*.

##### Lateral aspect

(Figures 7(c) and 8(c)) Cerotegument basically finely granular, larger granules on pedotectum I and in areas surrounding acetabula. Lateral sejugal furrow broad and deep, with dense granulation. Next to lateral border of bothridium triangular protrusion orientated caudally, slightly projecting above lateral sejugal furrow. Pedotectum I small rounded, slightly projecting. Pedotectum II absent. Lateral enantiophysis present, anterior projection triangular, well developed, posterior protrusion triangular and small. Discidium present, developed as strongly projecting triangular bulge between acetabulum III and IV.

##### Podosoma and venter

(Figures 7(b) and 8(b)) Cerotegument with larger granules next to acetabula and fine granules surrounding anal opening. Epimeral setation 1-0-1-1, all setae setiform and smooth, seta *1b* longest (approx. 45 µm), others significantly shorter (ca. 7 µm). Internal borders of all epimera well visible. A densely granulated median sternal cavity on epimeron I with slightly concave lateral borders resulting in inverted pear-like shape. Rounded genital plates with three pairs of fine setae (approx. 8 µm) arranged in longitudinal rows. Aggenital setae absent. Lyrifissure *iad* oblique adjacent to anterior corner of anal orifice. Outer part of preanal organ rectangular with rounded edges, inner part shaped like a transverse bar. Two pairs of short anal setae, *an_1-2_* and two pairs of short adanal setae, *ad_1-3_* (all approx. 8 µm).

##### Legs

**Figure 10.**
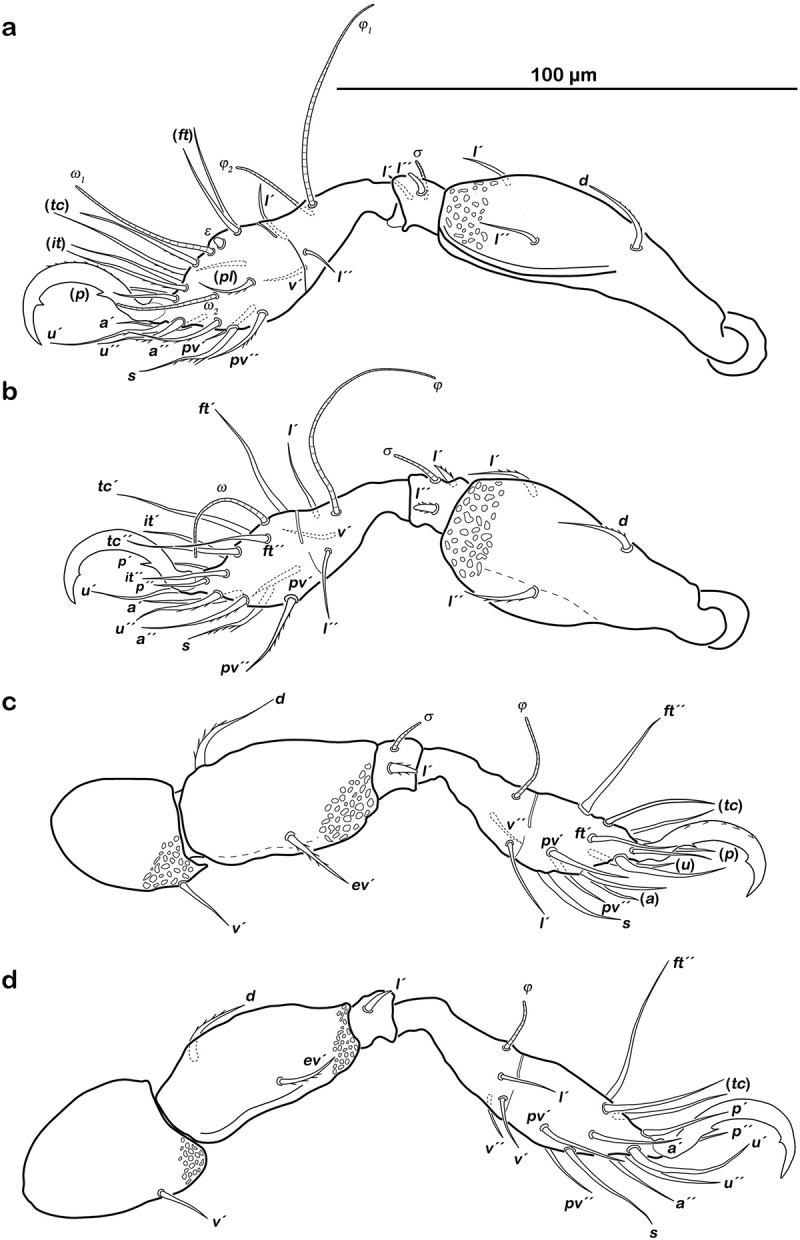
*Thalassozetes balboa* sp. nov. adult left legs, antiaxial view (a) leg I; (b) leg II; (c) leg III; (d) leg IV.

(Figure 10) Monodactylous. Long, strong hook-like claws, dorsally with slight dentation, hardly visible. Two ventral teeth on each claw, proximal one conspicuous and distal one weakly developed. Cerotegument generally finely granular, larger granules on all trochanters and femora. Femora with ventral carina. No porose areas discernible. Femoral setae serrate. Lateral setae of all genua thickened, blunt and slightly serrate. Ventral setae of tarsus serrate. Famulus *ε* developed as short broad knob. Solenidion *ω_2_* in lateral position. Chaetome and solenidia see Table 2.

#### Morphometrics

##### Univariate Statistics: Litoribates

The two *Litoribates* species *L. bonairensis* and *L. floridae* differ significantly in only 5 of the 15 measured variables (Table 3). The variability as indicated by cv is moderate (cv ≤0.10) in all characters.

**Table 3.** Univariate statistics for Caribbean *Litoribates* species.

	*L. bonairensis* (n = 19)					*L. floridae* sp. nov. (n = 16)					
	min	max	x	sd	cv	min	max	x	sd	cv	Mann–Whitney-*U*
*bl*	345	382	364.21	8.97	0.02	350	375	363.31	7.88	0.02	
*dPtI*	151	163	157.95	3.75	0.02	160	166	162.19	2.23	0.01	***
*db*	132	148	138.11	4.98	0.04	139	148	142.31	3.09	0.02	**
*ll*	46	65	51.95	5.16	0.10	52	65	59.44	4.16	0.07	***
*nw_c1_*	160	194	176.00	9.33	0.05	163	203	178.50	10.71	0.06	
*nw_da_*	228	252	239.58	7.83	0.03	231	259	244.25	8.63	0.04	
*nw_dm_*	219	259	237.84	10.54	0.04	222	259	237.13	9.49	0.04	
*cl*	89	108	99.58	5.63	0.06	92	108	100.06	4.57	0.05	
*cw*	80	89	84.53	2.41	0.03	83	86	83.94	1.44	0.02	
*dcg*	71	92	81.74	5.86	0.07	68	92	79.31	6.29	0.08	
*dac3*	129	142	135.47	3.66	0.03	135	142	138.63	3.14	0.02	*
*gl*	52	65	58.47	3.63	0.06	53	65	59.50	3.46	0.06	
*gw*	59	72	64.53	3.10	0.05	60	72	66.44	4.35	0.07	
*al*	80	86	81.26	1.82	0.02	80	86	83.81	1.94	0.02	***
*aw*	65	74	69.63	2.56	0.04	65	74	69.63	3.24	0.05	

Minimum (min), maximum (max), mean (x), standard deviation (sd), and coefficient of variation (cv), marked grey if equal or higher than 0.10. Results of Mann–Whitney-*U* test are given; *p < 0.05, **p < 0.01, ***p < 0.001.

##### Multivariate analyses: Litoribates

**Figure 11.**
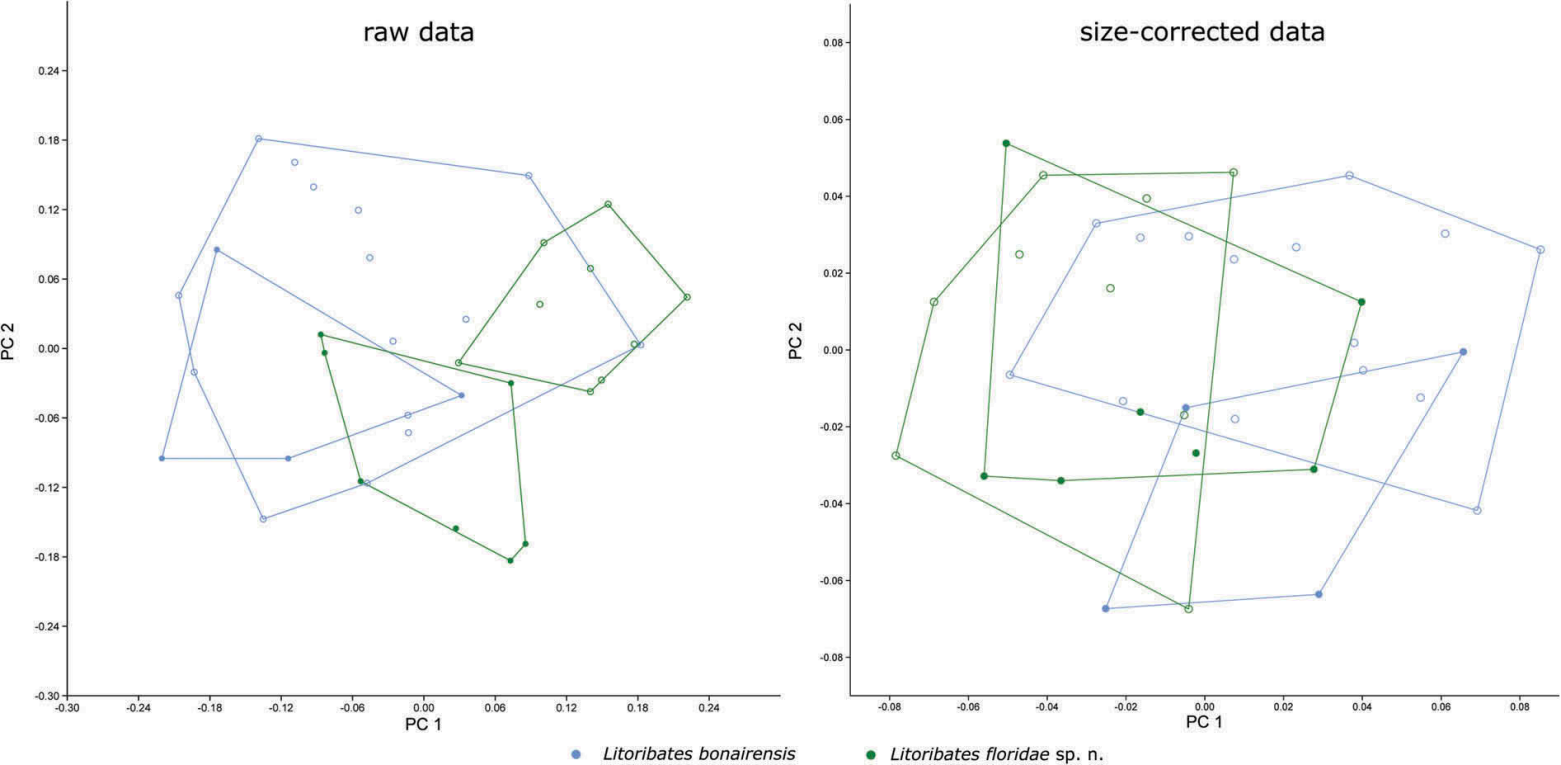
Scatter plots of PCA of *L. bonairensis* from Bonaire and *L. floridae* sp. nov. from Florida. Open symbols refer to females; filled symbols refer to male specimens. For better display of sexual dimorphism, each gender is given in a separate hull.

The two *Litoribates* species can be separated only partly by PCA on raw data and totally overlap in the PCA on size-corrected data (Figure 11). In the PCA on raw data, PC 1 (explaining 38.5% of the total variation) is responsible for the slight separation. The variable with by far the highest loading on PC 1, and thus contributing most to the separation, is the lenticulus length *ll* (Table 4). PC 1 also correlates slightly with “size” as defined by the geometric mean (*r* = 0.81), indicating that *L. floridae* individuals are slightly larger than those of *L. bonairensis*. The combination of PC 1 and PC 2 (explaining 25.0% of the total variation) reveals a sexual dimorphism. In *L. floridae*, males and females are clearly separated, while the two sexes overlap in *L. bonairensis*. The variable contributing the most to separation along PC 2 is the length of the genital opening *gl* (Table 4).

**Table 4.** Loadings of the two principal components PC 1 and PC 2 gained from PCA on data of *L. bonairensis* and *L. floridae* sp. nov.

	Raw data		Size-corrected data	
	PC 1	PC 2	PC 1	PC 2
*bl*	0.09	0.15	0.14	0.12
*dPtI*	0.13	0.10	0.03	−0.08
*db*	0.17	0.16	0.05	−0.09
*ll*	**0.79**	−0.44	**−0.67**	−0.11
*nw_c1_*	0.27	0.08	−0.37	−0.18
*nw_da_*	0.20	0.18	−0.03	0.13
*nw_dm_*	0.06	0.29	0.39	0.41
*cl*	0.18	0.12	−0.03	0.38
*cw*	0.03	0.12	0.12	0.00
*dcg*	−0.11	0.37	**0.42**	**−0.72**
*dac3*	0.11	0.15	0.08	−0.11
*gl*	0.21	**0.49**	0.12	0.15
*gw*	0.30	0.35	−0.03	0.19
*al*	0.11	−0.08	−0.08	0.03
*aw*	0.07	0.27	0.14	0.03

High loadings explaining differences between the species are given in bold.

Size correction resulted in a decrease of 87.1% of the total variation. After size correction, PCA no longer separates the two *Litoribates* species (Figure 11). The sexual dimorphism of *L. floridae* is not visible in the size-corrected data, but a slight separation of the sexes becomes apparent along PC 2 in *L. bonairensis*. The variable with the highest loading on PC 2 is *dcg* (Table 4).

The LDA on both raw and size-corrected data revealed that the two species are mainly separated by the variable *ll*, and the Hotelling’s T-test showed highly significant differences (*p* < 0.001) between them. In the raw data, 100% of the individuals can be correctly classified by all-samples LDA and 80% after leave-one-out cross-validation. After size correction, all-samples LDA still correctly classifies 97.14% and leave-one-out cross-validation LDA correctly classifies 74.29%.

##### Univariate Statistics: Thalassozetes

*Thalassozetes barbara* from Barbados and *T. balboa* from Panama differ significantly in 11 of the 20 measured variables (Table 5). The variability of almost all characters is moderate with a cv ≤0.10 in all populations, only *efw_2_* shows slightly higher values (cv between 0.11 and 013).

**Table 5.** Univariate statistics for Caribbean *Thalassozetes* species.

	*T. barabara* (n = 19)					*T. balboae* (n = 90)					
	min	max	x	sd	cv	min	max	x	sd	cv	Mann–Whitney-*U*
*bl*	271	302	284.68	8.63	0.03	277	312	292.58	9.34	0.03	**
*dPtI*	120	129	124.42	2.09	0.02	117	135	126.62	3.19	0.03	**
*db*	49	53	51.26	1.33	0.03	51	59	54.98	2.25	0.04	***
*ll*	28	34	30.53	2.29	0.08	31	46	38.97	3.61	0.09	***
*dnr*	31	43	36.58	2.65	0.07	28	40	34.00	2.84	0.08	***
*nw_da_*	154	169	158.89	5.02	0.03	142	182	161.86	7.33	0.05	
*nw_dm_*	163	182	170.32	5.62	0.03	154	194	173.50	7.17	0.04	
*nw_dp_*	145	169	154.37	5.79	0.04	142	175	156.83	6.27	0.04	
*cl*	74	83	77.16	2.54	0.03	70	83	78.58	2.50	0.03	*
*cw*	55	62	59.74	1.82	0.03	55	62	59.54	1.87	0.03	
*dre1*	105	114	110.68	2.43	0.02	108	123	115.67	2.96	0.03	***
*efl*	23	31	27.58	1.64	0.06	25	31	28.17	1.78	0.06	
*efw_1_*	22	31	25.47	2.29	0.09	19	30	23.78	2.04	0.09	**
*efw_2_*	19	25	21.11	2.28	0.11	14	22	16.57	2.07	0.13	***
*dcg*	68	77	72.32	2.50	0.03	69	81	75.30	2.41	0.03	***
*dac3*	92	105	96.11	2.92	0.03	92	108	99.83	3.23	0.03	***
*gl*	34	45	38.32	3.77	0.10	34	46	38.46	3.56	0.09	
*gw*	42	49	45.37	2.99	0.07	40	55	45.79	3.71	0.08	
*al*	62	68	63.26	1.82	0.03	60	71	64.16	2.16	0.03	
*aw*	46	52	49.11	1.79	0.04	43	55	48.90	2.23	0.05	

Minimum (min), maximum (max), mean (x), standard deviation (sd), and coefficient of variation (cv), marked grey if equal or higher than 0.10. Results of Mann–Whitney-*U* test are given; *p < 0.05, **p < 0.01, ***p < 0.001.

##### Multivariate analyses: Thalassozetes

**Figure 12.**
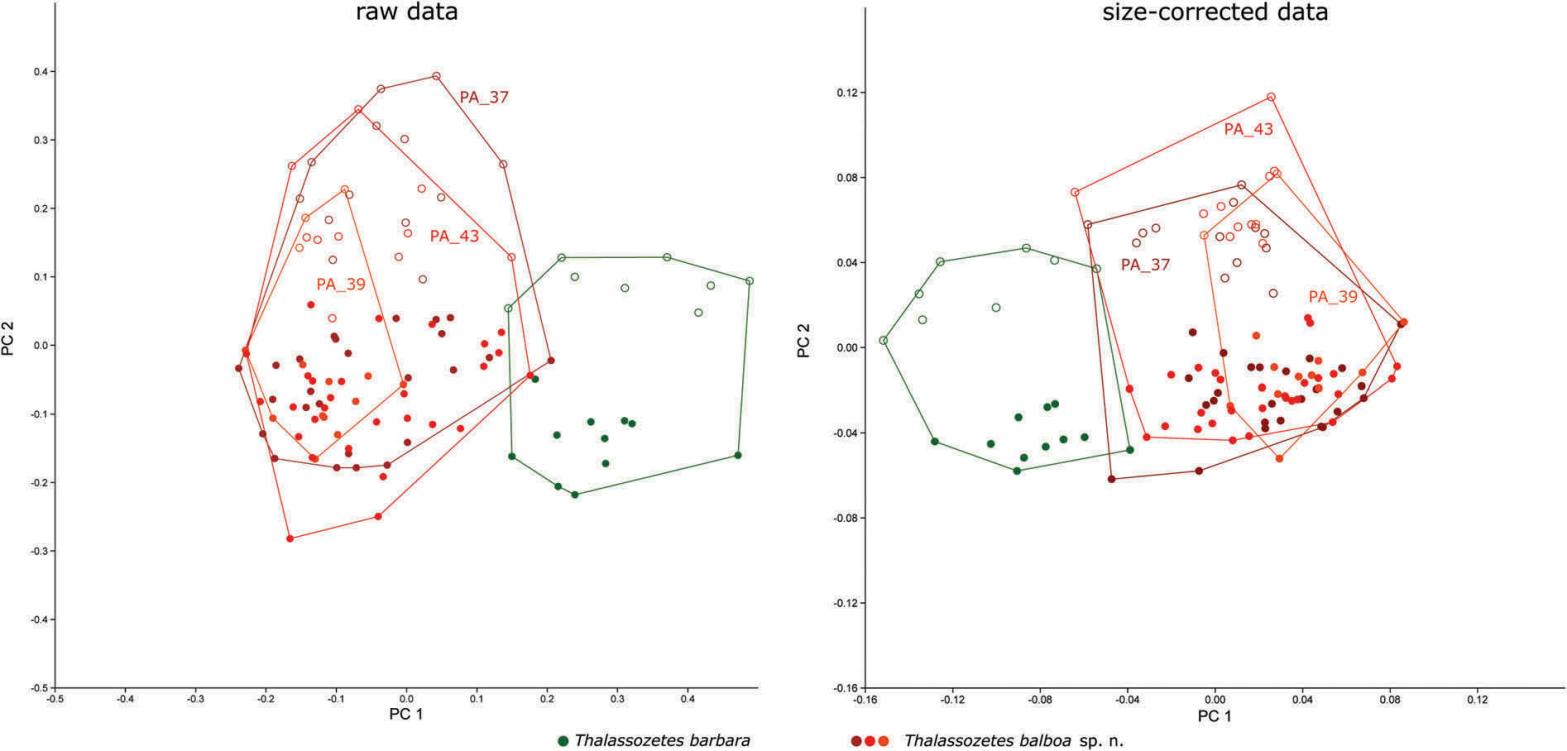
Scatter plots of PCA of *T. barbara* from Barbados and *T. balboa* sp. nov. from three different locations in Panama. Codes refer to sample sites given in the Materials and Method section; open symbols refer to females; filled symbols refer to male specimens.

In PCA on both raw and size-corrected data, PC 1 (explaining 33.6% of the total variation in raw data and 29.1% in size-corrected data) separated *T. barbara* from *T. balboa* with a very small overlapping area (Figure 12). The variation within *T. balboa* seems to be larger than that of *T. barbara*, and the larger variation is not caused by different sample sites as the three populations from Panama always overlap. In PCAs on both raw and size-corrected data, the variables with the highest loadings on PC 1 were *ll* and *efw2* (Table 6). A pronounced sexual dimorphism is revealed by PC 2 (explaining 25.6% and 17.0% of the total variation, respectively) in both raw and size-corrected data (Figure 12). The differences between the two sexes are more pronounced in *T. barbara* than in *T. balboa* PC 2 is strongly correlated with size (geometric mean) in the raw data (*r* = 0.96), indicating that females of both species are considerably larger than males. Variables contributing most to PC 2 were *gl* and *gw*, length and width of the genital opening. In the size-corrected data, there is only a weak correlation between PC 2 and size (*r* = 0.68). Here, the differences between the two sexes are again mainly caused by the variables *gl* and *gw*, which have the highest loadings for PC 2 (Table 6). The total variation decreased strongly, by 88.7%, after size correction.

**Table 6.** Loadings of the two principal components PC 1 and PC 2 gained from PCA on data of *T. barbara* and *T. balboa* sp. nov.

	Raw data		Size-corrected data	
	PC 1	PC 2	PC 1	PC 2
*bl*	−0.03	0.17	0.11	0.02
*dPtI*	−0.02	0.11	0.07	−0.11
*db*	−0.12	0.15	0.20	−0.04
*ll*	**−0.57**	0.30	**0.73**	0.14
*dnr*	0.16	0.18	−0.21	−0.13
*nw_da_*	−0.01	0.23	0.03	0.20
*nw_dm_*	−0.01	0.23	0.03	0.20
*nw_dp_*	0.00	0.19	0.01	0.07
*cl*	−0.03	0.06	0.08	−0.19
*cw*	0.03	0.10	−0.04	−0.13
*dre1*	−0.07	0.13	0.16	−0.06
*efl*	−0.02	−0.01	0.04	−0.28
*efw_1_*	0.22	0.17	−0.20	−0.05
*efw_2_*	**0.75**	0.17	**−0.46**	−0.15
*dcg*	−0.09	0.03	0.21	−0.31
*dac3*	−0.06	0.13	0.12	−0.06
*gl*	0.02	**0.54**	−0.10	**0.57**
*gw*	0.02	**0.46**	−0.08	**0.51**
*al*	−0.02	0.14	0.04	−0.05
*aw*	0.04	0.19	−0.05	0.06

High loadings explaining differences between the species are given in bold.

LDA showed again that separation of *T. barbara* from *T. balboa* was mainly caused by the variables *ll* and *efw2* in both raw and size-corrected data. All-samples LDA on raw data correctly classified 100% of all specimens and 96.3% after leave-one-out cross-validation. After size correction, the percentages of correctly classified individuals slightly dropped to 99.1% in all-samples LDA and increased to 97.3% after leave-one-out cross-validation, Hotelling’s T-test revealed highly significant differences between the two species.

#### Molecular genetic analyses

**Figure 13.**
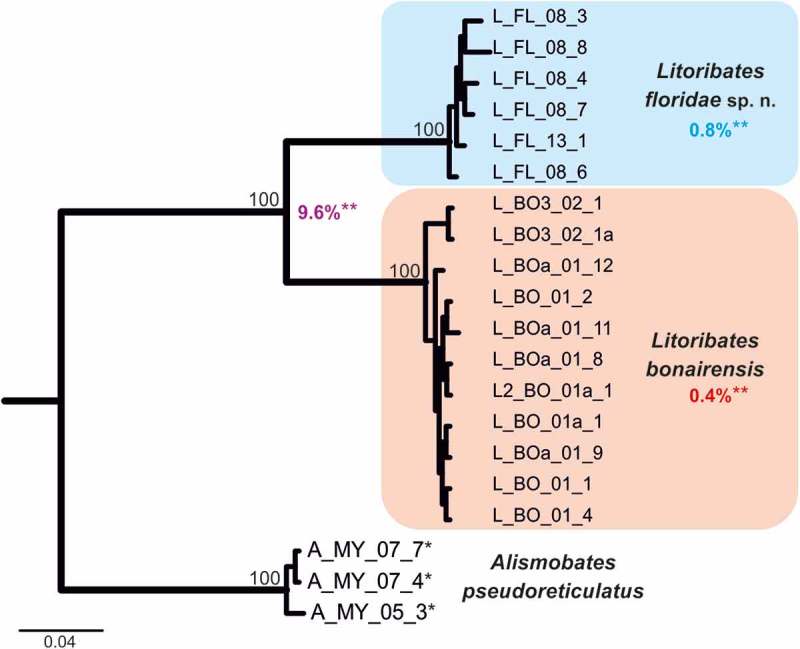
Bayesian inference tree of three fortuyniid species based on COI sequences constructed by means of MrBayes applying the GTR+I+G model. Posterior probabilities for main nodes are shown above branches. Sequences obtained from GenBank are marked by *. For details, see Table 1. ** Mean of intra- (blue, red) and interspecific (violet) uncorrected *p*-distances given in per cent.

To ensure the genetic distinctness of the investigated *Litoribates* species, classical barcoding by means of COI data was applied (Figure 13). The COI topology clearly separates *L*. *floridae* and *L. bonairensis*. The distinctness of these two species is underlined by a clear “barcoding gap” with high genetic differences. The highest intraspecific distance (uncorrected *p*-distance) amounted to 1.4% and the lowest interspecific distance to 9.6%.

**Figure 14.**
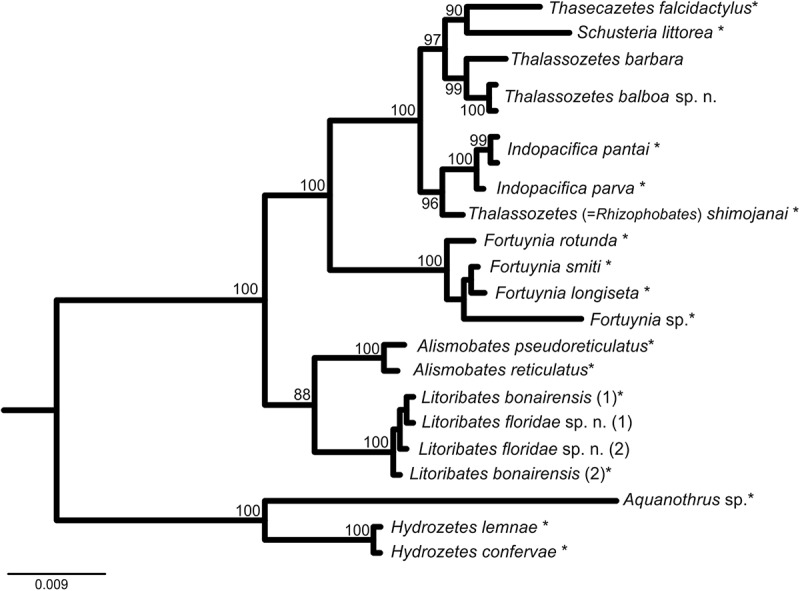
Bayesian inference tree of fortuyniid and selenoribatid taxa based on 18S rRNA sequences (1811 bp) constructed by means of MrBayes applying the GTR+I+G model. Posterior probabilities (>80) are shown near nodes. Sequences obtained from GenBank are marked by *. For details, see Table 1.

The 18S topology clearly demonstrates the monophyly of all investigated genera (Figure 14). Although the 18S rRNA gene is typically not suitable for species identification, *T. balboa* and *T. barbara* could be clearly separated with high statistical support. *Thalassozetes* species form the sister group of the *Schusteria* and *Thasecazetes* clade and together with the recently described *Indopacifica* species and *Rhizophobates* (=“*Thalassozetes shimojanai*,” for explanation of diverging taxon name, please refer to discussion) they build a monophyletic clade of Selenoribatidae. *Litoribates* spp. are placed as sister group of the Pacific *Alismobates* species; however, members of Fortuyniidae are shown to be paraphyletic with the genus *Fortuynia* being placed closer to Selenoribatidae.

## Discussion

### Systematics

**Figure 15.**
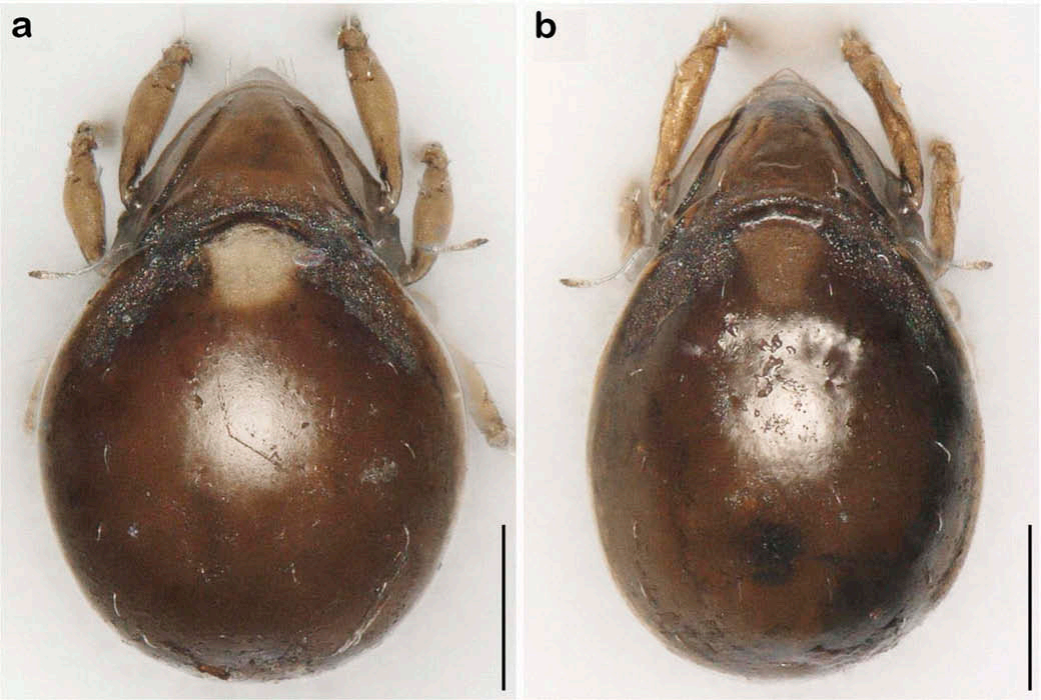
Photographs (stacked stereomicroscopic images) of female adult *Litoribates* specimens used for morphometric investigations. (a) *Litoribates floridae* sp. nov., Florida; (b) *Litoribates bonairensis*, Bonaire. Scale bars 100 µm.

*Litoribates floridae* sp. nov. and *L. bonairensis* have nearly identical morphology and represent cryptic species, which Knowlton (1993) defined as species that are difficult to distinguish using traditional morphology-based taxonomy. Apart from the irregular notogastral ridges in the humeral area being more pronounced in *L. floridae*, both species share all diagnostic characters and are difficult to distinguish under the microscope (see Figure 15). The morphometric analyses also demonstrate that the species are similar in size and shape, and only a few characters differ significantly between them. *Litoribates floridae* is basically slightly larger with a broader prodorsum (variables *dPtI* and *db*) and a longer lenticulus (*ll*); additionally, the sexual dimorphism is more pronounced in this species, with females having obviously larger genital openings than males. In contrast to morphology, genetic data clearly separate the species as shown by the large barcoding gap between the taxa; hence; the distinctness of the species is confirmed despite the striking morphological similarity.

We speculate that either of two evolutionary processes could be responsible for their near-identity. First, speciation may have been too recent for detectable morphological differences to have accumulated (Holland et al. 2004). Second, extreme or homogeneous environmental conditions may cause stabilizing selection, resulting in highly conserved morphologies (e.g. Bickford et al. 2007). The intertidal habitat represents an extreme environment, and both these intertidal species dwell in the same littoral habitat; therefore, stabilizing selection may well be responsible for their morphological similarity.

Within the genus *Thalassozetes, T. balboa* sp. nov. shares most characteristics with *T. barbara*. They differ only slightly, particularly in diverging notogastral surface structure, and numbers of adanal setae and of ventral teeth on the leg claws. Since the two species occupy the same geographic region, they may be derived from a common Caribbean ancestor. Molecular genetic data are not comprehensive enough to address this issue as there are no comparable data for the Mediterranean *T. riparius* and the Indo-Pacific *T. tenuisetosus*. In GenBank, there is a sequence presented under the name *Thalassozetes shimojanai* but this should be corrected to *Rhizophobates shimojanai* Karasawa & Aoki, 2005 (Pfingstl et al. 2018). The separation of *R. shimojanai* from true *Thalassozetes* species is further supported by their isolation in the phylogenetic tree based on 18S rRNA sequences (Figure 14).

### Ecology

The genus *Litoribates* has been associated mainly with mangrove leaf litter (Pfingstl et al. 2017), and the present study supports this generalization. All *L. floridae* sp. nov. specimens were found in high numbers in a mangrove leaf litter sample, except for one specimen found in a sample of algae growing on rock under a mangrove tree, which may have been accidental. The actual food source of *Litoribates* species, whether the decaying leaf litter itself or some other material, such as coccal algae, bacteria, or fungi, is not yet identified .

Members of the genus *Thalassozetes* also seem to be associated with specific coastal habitats. With one exception, there is a clear preference for intertidal rocky substrates, e.g. cliffs, rocks, and stones. The type species *T. riparius* was considered a characteristic inhabitant of the Mediterranean rocky littoral shore (Schuster 1963), *T. barbara* was suggested to prefer rocky habitats in the Caribbean area (Pfingstl et al. 2016), and *T. balboa* was predominantly found in algae growing on rocky substrate, with only four specimens collected from algae on mangrove roots. The exception is *T. tenuisetosus*, which was reported from intertidal algal sediment on a beach in India (Bayartogtokh and Chatterjee 2010). The specific food source used is not known for *T. riparius* and *T. tenuisetosus*, but *T. barbara* and *T. balboa* were observed to feed on the intertidal alga *Bostrychia*.

### Distribution

When the genus *Litoribates* was recorded for the first time in the Caribbean region, a wider distribution was anticipated, even though it was known from only Bonaire and the close Venezuelan coast (Pfingstl et al. 2017), and its trans-Caribbean distribution is now confirmed by our discovery of *L. floridae*. Their mangrove leaf litter habitat may facilitate wide dispersal as fallen leaves are easily washed into the sea and may drift on the surface over long distances.

The selenoribatid *T*. *balboa* sp. nov., on the other hand, was found in Panama and Florida, and despite the large distance between these areas, we suggest this species has a wide distribution along the Caribbean Central American coastline, with ongoing gene flow between the populations. Hydrochorous dispersal, i.e. drifting along ocean currents, has been considered the main mode of long distance transport for oribatid mites (e.g. Schatz 1991; Pfingstl 2013c), and in this case, the strong northward flowing Gulf Stream could facilitate dispersal along this vast shoreline. Since the Gulf of Mexico represents a large potential barrier, either *T. balboa* sp. nov. has colonized the whole Gulf coast or it has crossed the ocean from the Peninsula of Yucatan to Florida by drifting on the Gulf Stream. Presently, *T. balboa* sp. nov. seems to be restricted to the Western Caribbean, while *T. barbara* was only found in the Eastern Caribbean. As both species use the same habitat and food source, a syntopic occurrence or overlapping distribution areas seem rather unlikely.

## References

[CIT0001] Bayartogtokh B, Chatterjee T. 2010 Oribatid mites from marine littoral and freshwater habitats in India with remarks on world species of *Thalassozetes* (Acari: Oribatida). Zoological Studies. 49:839–854.

[CIT0002] Bickford D, Lohman DJ, Sodhi NS, Ng PKL, Meier R, Winker K, Ingram KK, Das I 2007 Cryptic species as a window on diversity and conservation. Trends in Ecology and Evolution. 22:148–155. 17129636 10.1016/j.tree.2006.11.004

[CIT0003] Casquet J, Thebaud C, Gillespie RG 2012 Chelex without boiling, a rapid and easy technique to obtain stable amplifiable DNA from small amounts of ethanol‐stored spiders. Molecular Ecology Resources. 12:136–141. 21943065 10.1111/j.1755-0998.2011.03073.x

[CIT0004] Dabert M, Witalinski W, Kazmierski A, Olszanowski Z, Dabert J 2010 Molecular phylogeny of acariform mites (Acari, Arachnida): strong conflict between phylogenetic signal and long-branch attraction artifacts. Molecular Phylogeny and Evolution. 56:222–241. 10.1016/j.ympev.2009.12.020 20060051

[CIT0005] Grandjean F 1953 Essai de classification des Oribates (Acariens). Bulletin de la Société zoologique de France. 78:421–446.

[CIT0006] Grandjean F. 1968 Schusteria littorea n.g., n.sp. et les Selenoribatidae (Oribates). Acarologia. 10:116–150.

[CIT0007] Holland BS, Dawson MN, Crow GL, Hofman DK 2004 Global phylogeography of *Cassiopea* (Scyphozoa: Rhizostomeae): molecular evidence for cryptic species and multiple invasions of the Hawaiian Islands. Marine Biology. 145:1119–1128.

[CIT0008] Iseki A, Karasawa S 2014 First record of *Maculobates* (Acari: Oribatida: Liebstadiidae) from Japan, with a redescription based on specimens from the Ryukyu Archipelago. Species Diversity. 19:59–69.

[CIT0009] Karasawa S, Aoki J 2005 Oribatid mites (Arachnida: Acari: Oribatida) from the marine littoral of the Ryukyu Archipelago, Southwestern Japan. Species Diversity. 10:209–233. 10.2108/zsj.24.1051 18088169

[CIT0010] Knowlton N 1993 Sibling species in the sea. Annual Review of Ecology and Systematics. 24:189–216.

[CIT0011] Krause A, Pachl P, Schulz G, Lehmitz R, Seniczak A, Schaefer I, Scheu S, Maraun M 2016 Convergent evolution of aquatic life by sexual and parthenogenetic oribatid mites. Experimental and Applied Acarology. 70:439–453. 27785647 10.1007/s10493-016-0089-3

[CIT0012] Luxton M. 1992 Oribatid mites from the marine littoral of Hong Kong (Acari: Cryptostigmata). In: Morton B, editor. The marine flora and fauna of Hong Kong and southern China III. Proceedings of the Fourth International marine Biological Workshop: The marine Flora and Fauna of Hong Kong and Southern China. Hong Kong: Kong Hong University Press p. 211–227.

[CIT0013] Hammen van der L 1960 *Fortuynia marina* gen. nov., sp. nov., an oribatid mite from the intertidal zone in Netherlands New Guinea. Zoologische Mededelingen. 37:1–9.

[CIT0014] Hammen van der L 1963 Description of *Fortuynia yunkeri* nov. spec., and notes on the Fortuyniidae nov. fam. Acarologia. 5:152–167.

[CIT0015] Hammer ØH, Harper DAT, Ryan PD 2001 PAST: paleontological statistics software package for education and data analysis. Palaeontologica Electronica. 4:1–9.

[CIT0016] Norton RA, Behan-Pelletier VM 2009 Suborder Oribatida Chapter 15. In: Krantz G, DE W, editors. A manual of acarology. Lubbock: Texas Tech University Press p. 430–564.

[CIT0017] Otto JC Wilson K 2001 Assessment of the usefulness of ribosomal 18S and mitochondrial COI sequences in Prostigmata phylogeny In: Halliday RB, Walter DE, Proctor H, Norton RA, Colloff MJ, editors. Acarology: proceedings of the 10th International Congress. Melbourne: CSIRO Publisher p. 100–109.

[CIT0018] Pepato AR, Da Rocha CE, Dunlop JA 2010 Phylogenetic position of the acariform mites: sensitivity to homology assessment under total evidence. BMC Evolutionary Biology. 10:235. 20678229 10.1186/1471-2148-10-235 PMC2933639

[CIT0019] Pfingstl T 2013a Habitat use, feeding and reproductive traits of rocky-shore intertidal mites from Bermuda (Oribatida: Fortuyniidae and Selenoribatidae). Acarologia. 53:369–382.

[CIT0020] Pfingstl T 2013b *Thalassozetes barbara* sp. n. (Acari, Oribatida), a new intertidal species from the coast of Barbados. Acarologia. 53:417–424.

[CIT0021] Pfingstl T 2013c Resistance to fresh and salt water in intertidal mites (Acari: Oribatida): implications for ecology and hydrochorous dispersal. Experimental and Applied Acarology. 61:87–96. 23456607 10.1007/s10493-013-9681-y PMC3742429

[CIT0022] Pfingstl T 2017 The marine-associated lifestyle of ameronothroid mites (Acari, Oribatida) and its evolutionary origin: a review. Acarologia. 57:693–721.

[CIT0023] Pfingstl T, Baumann J 2017 Morphological diversification among island populations of intertidal mites (Acari, Oribatida, Fortuyniidae) from the Galápagos archipelago. Experimental and Applied Acarology. 72:115–131. 28634717 10.1007/s10493-017-0149-3 PMC5486844

[CIT0024] Pfingstl T, Baumann J, Lienhard A, Schatz H 2017 New Fortuyniidae and Selenoribatidae (Acari, Oribatida) from Bonaire (Lesser Antilles) and morphometric comparison between Eastern Pacific and Caribbean populations of Fortuyniidae. Systematic and Applied Acarology. 22:2190–2217.

[CIT0025] Pfingstl T, De Los Santos G, Lienhard A 2016 First records of intertidal mite species (Acari: Acariformes: Oribatida) from Hispaniola’s coasts with two new records for the Caribbean. Revista Ibérica De Aracnologica. 29:41–44.

[CIT0026] Pfingstl T, Krisper G 2014 Plastron respiration in marine intertidal oribatid mites (Acari, Fortuyniidae and Selenoribatidae). Zoomorphology. 133:359–378.

[CIT0027] Pfingstl T, Lienhard A 2017 *Schusteria marina* sp. nov. (Acari, Oribatida, Selenoribatidae) an intertidal mite from Caribbean coasts, with remarks on taxonomy, biogeography and ecology. International Journal of Acarology. 43:462–467. 28931960 10.1080/01647954.2017.1348393 PMC5586027

[CIT0028] Pfingstl T, Lienhard A, Jagersbacher-Baumann J 2014 Hidden in the mangrove forest: the cryptic intertidal mite *Carinozetes mangrovi* sp. nov. (Acari, Oribatida, Selenoribatidae). Experimental and Applied Acarology. 63:481–495. 24687175 10.1007/s10493-014-9802-2

[CIT0029] Pfingstl T, Lienhard A, Shimano S, Yasin ZB, Shau-Hwai AT, Jantarit S, Petcharad B 2018 Systematics, genetics and biogeography of intertidal mites (Acari, Oribatida) from the Andaman Sea and Strait of Malacca. Journal of Zoological Systematics and Evolutionary Research. doi:10.1111/jzs.12244 PMC6378605 30828135

[CIT0030] Pfingstl T, Schatz H 2017 New littoral mite species (Acari, Oribatida, Fortuyniidae) from the Galápagos archipelago with ecological and zoogeographical considerations. Zootaxa. 4244:39–64. 28610129 10.11646/zootaxa.4244.1.2

[CIT0031] Pfingstl T, Schuster R 2012 Carinozetes nov. Gen. (Acari: Oribatida) from Bermuda and remarks on the present status of the Family Selenoribatidae. Acarologia. 52:377–409.

[CIT0032] Pfingstl T, Schuster R 2014 Global distribution of the thalassobiontic Fortuyniidae and Selenoribatidae (Acari, Oribatida). Soil Organisms. 86:125–130.

[CIT0033] Pugh PJA, King PE, Fordy MR 1990 Respiration in *Fortuynia maculata* Luxton (Fortuyniidae: Cryptostigmata: Acarina) with particular reference to the role of van der Hammen’s organ. Journal of Natural History. 24:1529–1547.

[CIT0034] Rambaut A, Drummond A 2007 Tracer v1.4; [cited 2018 Jun 20]. Available from: http://beast.bio.ed.ac.uk/Tracer

[CIT0035] Ronquist F, Huelsenbeck JP 2003 MrBayes 3: Bayesian phylogenetic inference under mixed models. Bioinformatics. 19:1572–1574. 12912839 10.1093/bioinformatics/btg180

[CIT0036] Schäffer S, Krisper G, Pfingstl T, Sturmbauer C 2008 Description of *Scutovertex pileatus* sp. nov. (Acari, Oribatida, Scutoverticidae) and molecular phylogenetic investigation of congeneric species in Austria. Zoologischer Anzeiger. 247:249–258.

[CIT0037] Schatz H 1991 Arrival and establishment of Acari on oceanic islands In: Dusbábek F, Bukva V, editors. Modern acarology. Vol. 2 Prague: Academia Prague and SPB Academic Publishing, The Hague p. 613–618

[CIT0038] Schuster R 1963 *Thalassozetes riparius* n. gen., sp. n., eine litoralbewohnende Oribatide von bemerkenswerter morphologischer Variabilität (Acari-Oribatei). Zoologischer Anzeiger. 171:391–403.

[CIT0039] Schuster R 1966 Hornmilben (Oribatei) als Bewohner des marinen Litorals. Veröffentlichungen des Institutes für Meeresforschung Bremerhaven. Sonderband. II:319–327.

[CIT0040] Schuster R 1977 Die Selenoribatidae, eine thalassobionte Familie der Hornmilben. Acarologia. 19:155–160.

[CIT0041] Schuster R 1989 Transoceanic distribution of air-breathing littoral mites. Progress in Acarology. 1:355–362.

[CIT0042] Skoracka A, Dabert M 2010 The cereal rust mite *Abacarus hystrix* (Acari: Eriophyoidea) is a complex of species: evidence from mitochondrial and nuclear DNA sequences. Bulletin of Entomological Research. 100:263–272. 19671206 10.1017/S0007485309990216

[CIT0043] Strenzke K. 1961 Selenoribates foveiventris n. gen., n. sp., aus der unterirdischen Feuchtzone der Küste des Roten Meeres (Acarina: Oribatei). Kieler Meeresforschung. 17:89–93.

[CIT0044] Tamura K, Stecher G, Peterson D, Filipski A, Kumar S 2013 MEGA6: molecular evolutionary genetics analysis version 6.0. Molecular Biology and Evolution. 30:2725–2729. 24132122 10.1093/molbev/mst197 PMC3840312

